# Melatonin Inhibits Dengue Virus Infection via the Sirtuin 1-Mediated Interferon Pathway

**DOI:** 10.3390/v13040659

**Published:** 2021-04-11

**Authors:** Atthapan Morchang, Shilu Malakar, Kanchanaphan Poonudom, Sansanee Noisakran, Pa-thai Yenchitsomanus, Thawornchai Limjindaporn

**Affiliations:** 1 Division of Molecular Medicine, Department of Research and Development, Faculty of Medicine Siriraj Hospital, Mahidol University, Bangkok 10700, Thailand; atthapan.mor@hotmail.com (A.M.); ptyench@gmail.com (P.-t.Y.); 2 Department of Anatomy, Faculty of Medicine Siriraj Hospital, Mahidol University, Bangkok 10700, Thailand; me_shilum@yahoo.com (S.M.); kan.poonudom@gmail.com (K.P.); 3 Molecular Biology of Dengue and Flaviviruses Research Team, Medical Molecular Biotechnology Research Group, National Center for Genetic Engineering and Biotechnology, National Science and Technology Development Agency, Bangkok 10700, Thailand; snoisakran@yahoo.com; 4 Division of Dengue Hemorrhagic Fever Research, Research Department, Faculty of Medicine Siriraj Hospital, Mahidol University, Bangkok 10700, Thailand; 5 Siriraj Center of Research Excellence in Dengue and Emerging Pathogens, Faculty of Medicine Siriraj Hospital, Mahidol University, Bangkok 10700, Thailand

**Keywords:** melatonin, dengue virus infection, DENV, sirtuin 1 (SIRT1)-mediated interferon (IFN) pathway

## Abstract

Dengue virus (DENV) is the causative pathogen in the life-threatening dengue hemorrhagic fever and dengue shock syndrome. DENV is transmitted to humans via the bite of an infected *Aedes* mosquito. Approximately 100 million people are infected annually worldwide, and most of those live in tropical and subtropical areas. There is still no effective drug or vaccine for treatment of DENV infection. In this study, we set forth to investigate the effect of melatonin, which is a natural hormone with multiple pharmacological functions, against DENV infection. Treatment with subtoxic doses of melatonin dose-dependently inhibited DENV production. Cross-protection across serotypes and various cell types was also observed. Time-of-addition assay suggested that melatonin exerts its influence during the post-entry step of viral infection. The antiviral activity of melatonin partly originates from activation of the sirtuin pathway since co-treatment with melatonin and the sirtuin 1 (SIRT1) inhibitor reversed the effect of melatonin treatment alone. Moreover, melatonin could modulate the transcription of antiviral genes that aid in suppression of DENV production. This antiviral mechanism of melatonin suggests a possible new strategy for treating DENV infection.

## 1. Introduction

DENV is a mosquito-borne virus belonging to the family *Flaviviridae* and genus *Flavivirus*. Based on antigenicity, the virus is classified into four distinct serotypes, including DENV serotypes 1–4 (DENV1–4). DENV is enveloped and composed of a single-stranded positive-sense RNA genome of approximately 10.9 kilobases, which encodes a polyprotein that is processed by the host and viral proteases into three structural proteins; capsid (C), envelope (E) and membrane protein (M), and seven non-structural (NS) proteins. The viral replication begins with a receptor-mediated endocytosis, and the virus uses the host cell’s machinery to replicate its genome and proteins [1]. While primary infection often results in asymptomatic or mild dengue fever (DF), the more severe forms of the disease manifest as dengue hemorrhagic disease (DHF) or dengue shock syndrome (DSS) [2]. DHF is characterized by vascular leakage and hypovolemic shock, and it often develops in secondary infection due to antibody-dependent enhancement (ADE) [3]. DENV infection continues to be a persistent public health problem because there is still no effective treatment for this disease. Current data estimates that there are approximately 390 million human infections per year, and that Asia shoulders most of the burden of DENV infection [4]. The development of a vaccine against this viral infection has been a challenge due to the complexity of the virus serotypes, as well as the complication of ADE during the infection [5]. The absence of both a reliable animal model, and a reliable marker for immunity detection, further obstruct the development of an effective therapy for this disease.

Many studies have been conducted, or are ongoing, which identify potent anti-DENV agents. Moreover, multiple strategies have been investigated, including enzyme-based screening, viral replication-based screening, structure-based rational design, virtual screening, and fragment-based screening; however, all of these techniques have a lag from bench to bedside [6]. Via the adoption of the drug repurposing approach, several drugs, including balapiravir, chloroquine, celgosivir, and lovastatin, were identified as anti-DENV drugs [7]. Unfortunately, clinical trials revealed that all of these drugs have insufficient clinical efficacy. The discovery of a novel effective drug against DENV infection is, therefore, urgently needed, and drug repurposing may be a strategy that expedites the discovery of a drug that can effectively treat DENV infection. Accordingly, in this study, we set forth to investigate the effect of melatonin against DENV infection.

Melatonin (MEL) is a pleiotropic peptide hormone that is naturally secreted from the pineal gland to regulate the circadian rhythm [8]. The dark-light cycle between night and day stimulates the secretion of melatonin, which induces the sleep-wake cycle in humans. Melatonin is used as a supplement remedy for sleep-related disorders, such as jet lag, insomnia, and sleep deprivation. Interestingly, melatonin has emerged as a potential drug candidate for treating viral infections, including SARS CoV-2 infection [9]. The hepatoprotective role of melatonin was also studied in an animal model of fulminant hepatic failure [10]. Melatonin reduced apoptosis, necroptosis, mitochondrial permeability transition pore (mPTP) -driven cell death, and autophagy [11]. Among other cellular pathways, melatonin is known to increase the expression of sirtuin 1 (SIRT1) in different experimental conditions [12,13]. A recent study showed that SIRT1-mediated inhibition of high mobility group box 1 (HMGB1) translocation suppresses DENV replication via elicitation of interferon (IFN)-stimulated genes (ISGs) [14]. ISGs are effector molecules of the IFN signaling pathway, which is a crucial component of the innate immune response of the host to restrict DENV replication. Host-pathogen recognition leads to the production of IFN, which triggers a signaling cascade involving activation of the Janus kinase (JAK) protein tyrosine kinases, signal transducer and activator of transcription (STAT) phosphorylation, and ISGs. IFNs do not have the ability to directly inhibit virus replication; however, they activate hundreds of ISGs to induce a significant antiviral state [15].

In the current study, we investigated the effect of melatonin on DENV infection, its influence on the lifecycle of the virus, and its possible molecular mechanism of action. We found that melatonin inhibited DENV production most probably in the early phase of DENV replication. This antiviral activity of melatonin is likely originated from the activation of the SIRT1 pathway via the elicitation of ISGs.

## 2. Materials and Methods

### 2.1. Cell Lines and Virus

Huh7 human hepatoma cells and A549 human lung epithelial cells were cultured in Dulbecco’s Modified Eagle Medium (DMEM) (Gibco; Invitrogen, Carlsbad, CA, USA). Ea.hy.926 human endothelial-like cells were cultured in DMEM/Nutrient Mixture F-12 (DMEM/F-12) (Gibco; Invitrogen, Carlsbad, CA, USA). U937 human monocyte cells were cultured in RPMI Medium (, USA). Vero cells, which are African green monkey kidney cells, were cultured in Minimum Essential Medium (MEM) (Gibco; Invitrogen, Carlsbad, CA, USA). *Aedes albopictus* cells (C6/36) were cultured in L-15 media (Gibco; Invitrogen, Carlsbad, CA, USA). Cell lines were cultured in their respective media, supplemented with 10% heat-inactivate fetal bovine serum (FBS, Gibco; Invitrogen, Carlsbad, CA, USA), 100 U/mL penicillin, and 100 μg/mL streptomycin (Sigma, St. Louis, MO, USA). Leibovitz’s L-15 media was additionally supplemented with 10% tryptose phosphate broth (TPB). Cell lines were maintained in a humidified, 37 °C, 5% CO_2_ incubator, except for the C6/36 cells, which were maintained in a 28 °C incubator. DENV1 (strain Hawaii), DENV2 (strain 16681), DENV3 (strain H-87), and DENV4 (strain H241) were propagated in C6/36 cells and quantified by focus-forming unit (FFU) assay.

### 2.2. Chemical Compounds

MEL, N-acetyl cysteine (NAC), and the SIRT1 inhibitor (EX-527) were purchased from Sigma-Aldrich Corporation (St. Louis, MO, USA). The compounds were dissolved in dimethyl sulfoxide (DMSO) (Sigma-Aldrich Corporation, St. Louis, MO, USA) under sterile conditions to create a 100 mM stock solution, which was maintained at −20 °C until use. To obtain a working solution, the stock solution was diluted in fresh complete media before treatment. The concentration of DMSO, which was used as the vehicle control, did not exceed 0.4% *v*/*v* throughout the study.

### 2.3. DENV Infection and MEL Treatment

Cell lines were infected with DENV at multiplicity of infection (MOI) 1 and allowed to adsorb for 2 h. Unbound viruses were washed before proceeding with the experiments. Cells without infection were used as mock control and DMSO was used as a vehicle control.

### 2.4. Cell Viability Assay

Cell viability was determined using PrestoBlue^TM^ Cell Viability Assay. Briefly, 0.03 × 10^6^ Huh7 cells were seeded in a 96-well plate and treated with various concentrations of MEL or NAC in a total volume of 100 µL. After 24 h, 10 µL of PrestoBlue^TM^ (Thermo Fisher Scientific, Waltham, MA, USA) reagent was added into the well and incubated for 30 min in a 37 °C incubator. The fluorescent signal was then detected at an excitation/emission wavelength of 560/590 nm using a Synergy^TM^ Mx Microplate Reader (BioTek Instruments, Inc., Winooski, VT, USA). Cell viability was calculated as the percentage of untreated cells.

### 2.5. Focus-Forming Unit (FFU) Assay

Virus supernatants collected from untreated and compound-treated cells were titrated by FFU assay. Briefly, 0.02 × 10^6^ Vero cells were seeded in 96-well culture plate. The virus supernatant was ten-fold diluted in 2% FBS MEM ranging from 10^−1^ to 10^−7^. The cells were infected with the diluted virus for 2 h. Overlay media 1.5% carboxy methyl cellulose (CMC), 10% FBS MEM was added and incubated for 72 h in a humidified, 37 °C, 5% CO_2_ incubator. The cells were then washed with phosphate-buffered saline (PBS) solution, fixed with 3.8% formaldehyde, and permeabilized with 0.1% Triton-X 100 (each step for 20 min). The cells were incubated with mouse monoclonal anti-DENV E antibody (4G2) produced from a previously established hybridoma cell [16], this was used without dilution for 1 h at 37 °C, followed by horseradish peroxidase (HRP)-conjugated rabbit anti-mouse secondary antibody (1:1000) (Dako, Denmark) for 30 min at 37 °C. Foci were stained using 3–3′ diaminobenzidine (DAB) solution containing NiCl_2_ and H_2_O_2_. The foci were counted under a light microscope, and the virus titer was calculated as FFU/mL.

### 2.6. Time-of-Addition Assay

Time course treatment was performed at pre-during, and post-infection. For pre-infection treatment, Huh7 cells were treated with MEL for 3 h, and the cells were washed with PBS before infection. For during infection treatment, the cells were simultaneously incubated with the virus and MEL for 2 h and then washed with PBS before being replenished with complete media. For post-infection treatment, after the cells were infected with the virus for 2 h, the cells were washed with PBS and immediately replenished with complete media containing MEL (+2 h) for 5, 8, and 14 h (+5, +8, and +14 h). In all treatment conditions, the supernatants were collected after 24 h post-infection (h pi). The supernatants were then titrated by FFU assay.

### 2.7. Real-Time Reverse Transcription Polymerase Chain Reaction (RT-PCR) for mRNA Expression

Total RNA was isolated using TRIzol^TM^ Reagent (Invitrogen, Carlsbad, CA, USA), following the manufacturer’s instructions. RNA concentration and purity were measured using a Nanodrop Spectrophotometer (Thermo Fisher Scientific, Waltham, MA, USA). One microgram of RNA from each sample was reverse-transcribed into complementary DNA (cDNA) using a SuperScript^TM^ III First-Strand Synthesis System (Thermo Fisher Scientific, Waltham, MA, USA). cDNA was used as the template for detection of gene expression using LightCycler^®^ 480 SYBR Green I Master (Roche Applied Science, Penzberg, Germany), and the gene-specific primer pairs that were used are listed in Table 1. Real-time RT-PCR was performed on a LightCycler^®^ 480 Instrument II (Roche Applied Science) under the following temperature conditions: pre-incubation at 95 °C for 10 min, followed by 45 cycles of denaturation at 95 °C for 10 s, annealing at 60 °C for 10 s, and extension at 72 °C for 20 s. Relative gene expression was calculated according to 2^−ΔΔCt^ values compared between test and control samples. Glyceraldehyde 3-phosphate dehydrogenase (GAPDH) was used as a housekeeping gene.

### 2.8. Immunofluorescence Assay (IFA) for Detection of Viral Protein

Cells were seeded, infected, and treated on a glass cover slip. The cells were fixed with 4% paraformaldehyde for 20 min, followed by permeabilization with 0.2% Triton-X for 10 min at room temperature. The cells were then incubated with either mouse monoclonal anti-DENV E antibody (4G2), or, anti-DENV NS1 antibody produced from previously established hybridoma cell [17] were used without dilution for 60 min at 37 °C. The cells were washed and incubated with secondary antibodies (rabbit anti-mouse IgG-Alexa 488) (1:500) (Dako, Germany) and nuclear-staining dye (Hoechst 33342) (1:1000) for 60 min at 37 °C in the dark. After washing, the cover slips were mounted onto a glass slide using 50% glycerol. A fluorescent image was captured using an LSM 510 Meta Confocal Microscope (Carl Zeiss AG, Jena, Germany).

### 2.9. Western Blot Analysis for SIRT1 Protein Expression

Total protein was collected from mock or DENV-infected Huh7 cells treated with MEL with and without EX-527. 50 µg of total protein was used for sodium dodecyl sulfate-polyacrylamide gel electrophoresis (SDS-PAGE) and blotted onto a nitrocellulose membrane. The membrane was blocked with 5% skim milk and incubated overnight with a mouse monoclonal anti-SIRT1 antibody (B-10) (1:200) (sc-74504, Dallas, TX, USA) and a mouse monoclonal anti-GAPDH antibody (0411) (1:1000) (sc-47724, Santa Cruz Biotechnology, Dallas, TX, USA). The membrane was then incubated with an HRP-conjugated rabbit anti-mouse secondary antibody (1:1000) (Dako, Denmark) in room temperature for 1 h. The protein bands were detected by chemiluminescence substrate (SuperSignal West Pico Chemiluminescent Substrate; Thermo Fisher Scientific, MA, USA). The protein band from each sample was normalized with GAPDH. The result is shown in Supplementary Figure S2.

### 2.10. Statistical Analysis

Data acquired from three independent experiments are presented as mean ± standard error of the mean (SEM). The student’s *t*-test was performed using GraphPad Prism version 5 (GraphPad Software, Inc., San Diego, CA, USA). An asterisk sign (*) denotes statistical significance at a *p*-value of less than 0.05 (*p* < 0.05).

## 3. Results

### 3.1. MEL Inhibits DENV Production

**Figure 1:**
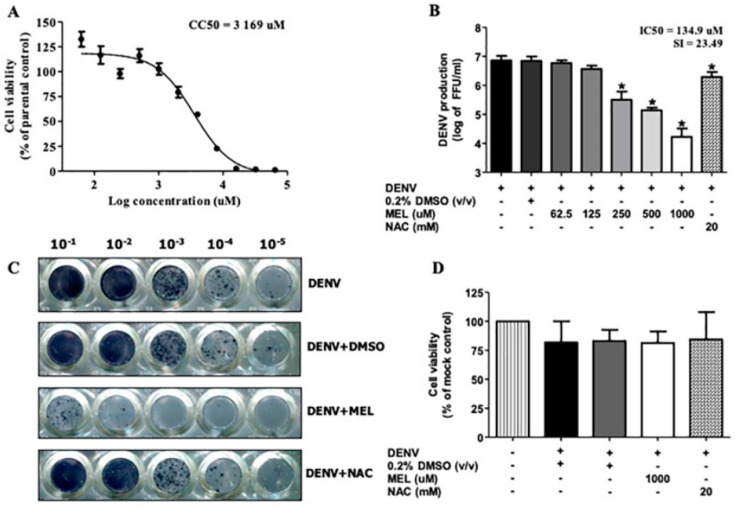
Antiviral effect of melatonin (MEL) on dengue virus (DENV2)-infected Huh7 cells. (**A**) Huh7 cells were inoculated with MEL in a 2-fold serial dilution for 24 h. Cell viability was measured by Presto Blue assay and the result was plotted into a graph, displaying the cytotoxic concentration (CC_50_). (**B**) Huh7 cells were infected with DENV2 at multiplicity of infection (MOI) 1 and treated with MEL at various concentrations. Culture supernatant were collected at 24 h pi and FFU assay was performed. (**C**) Representative picture for focus-forming unit (FFU) assay from one of the three independent experiments using 1000 µM MEL and 20 mM N-acetyl cysteine (NAC). (**D**) Cell viability was checked for the same time point. The asterisk (*) sign denotes *p* < 0.05 considered to be statistically significant.

To determine the subtoxic dose of MEL, 50% cytotoxic concentration (CC_50_) was obtained by cell viability assay. At 24 h post-treatment, the CC_50_ value of MEL in Huh7 cells was 3169 µM (Figure 1A), thus, the subtoxic doses of 1000, 500, 250, 125, and 62.5 µM were selected for subsequent experiments. To investigate the anti-DENV effect of MEL, the virus titer from DENV2-infected Huh7 cells, both with and without MEL treatment, was compared at 24 h pi. Treatment with MEL exhibited dose-dependent inhibition of DENV production in Huh7 cells (Figure 1B). MEL treatment at 1000, 500, and 250 µM reduced the virus titer from 7.21 × 10^6^ to 1.66 × 10^4^, 1.36 × 10^5^, and 3.16 × 10^5^ FFU/mL, respectively. The half maximal inhibitory concentration (IC_50_) was 134.9 µM and selective index (SI) was 23.49. In addition, the inhibitory effect of MEL was stronger than that of NAC which reduced the viral titer to 1.9 × 10^6^ FFU/mL. NAC showed protective activity in fulminant hepatic failure condition and decreased DENV replication in in vitro and in vivo model of DENV infection [18,19]. The foci representative of virus titer in Figure 1C clearly demonstrates the anti-DENV activity of MEL at 1000 µM concentration. This effect was not interfered by cell death, since no significant change of viability was observed among untreated and treated cells (more than 80% in all groups) (Figure 1D).

### 3.2. MEL Exhibits Anti-DENV Activity in All Serotypes

**Figure 2:**
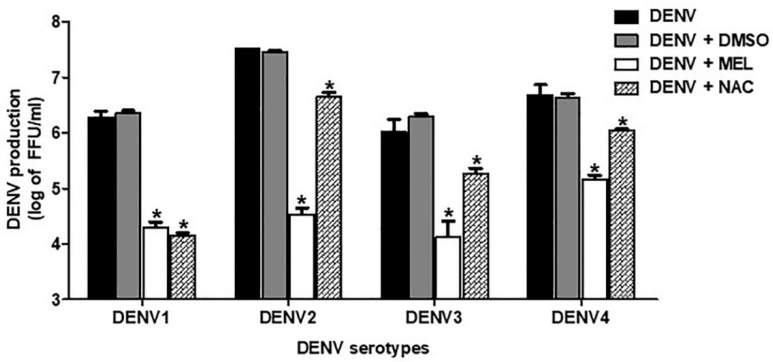
Antiviral effect of MEL on DENV serotypes. Huh7 cells were infected with DENV serotype 1, 2, 3, and 4 at MOI 1 and treated with vehicle control or 1000 µM MEL or 20 mM NAC. Culture supernatant were collected at 24 h and FFU assay was done. The asterisk (*) sign denotes *p* < 0.05 considered to be statistically significant.

We investigated the inhibitory effect of MEL on DENV-infected Huh7 cells in all serotypes of DENV. Our results showed that MEL is capable of reducing DENV production in all DENV serotypes, though the inhibitory level varied among serotypes (Figure 2). MEL treatment reduced the DENV1 titer from 1.93 × 10^6^ to 2.0 × 10^5^ FFU/mL, the DENV2 titer from 3.21 × 10^7^ to 3.33 × 10^4^ FFU/mL, the DENV3 titer from 1.3 × 10^6^ to 1.33 × 10^4^ FFU/mL, and the DENV4 titer from 4.33 × 10^6^ to 1.46 × 10^5^ FFU/mL.

### 3.3. MEL Exhibits Anti-DENV Activity in Different Cell Lines

**Figure 3:**
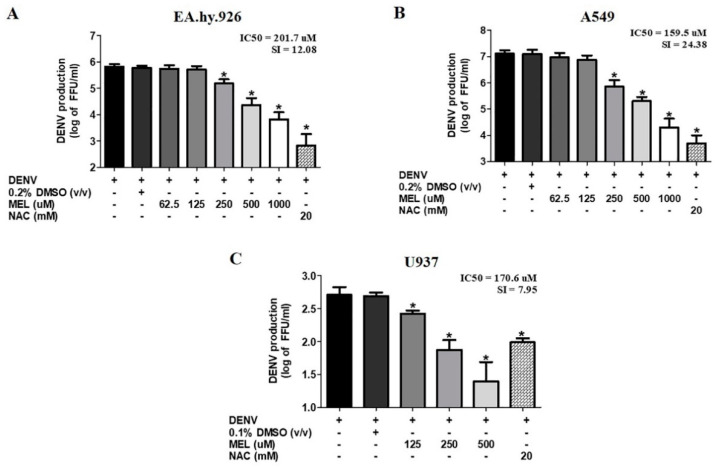
Antiviral effect of MEL in different cell lines. (**A**) EA.hy.926, (**B**) A549, and (**C**) U937 cells were infected with DENV2 at MOI 1 and treated with vehicle control or various concentrations of MEL or 20 mM NAC. Culture supernatant were collected at 24 h pi and FFU assay was done. The asterisk (*) sign denotes *p* < 0.05 considered to be statistically significant.

In addition to hepatic cell line, important target cells for DENV infection include lung, endothelial, and immune cells. To investigate the effect of MEL on these cells, DENV-infected A549, EA.hy.926, and U937 cells were treated with subtoxic doses of MEL for 24 h. Similar to that which was observed in Huh7 cells, treatment with MEL significantly inhibited DENV production in EA.hy.926 cells with an IC_50_ of 201.7 μM and an SI of 12.08 (Figure 3A); A549 cells with an IC_50_ of 159.5 μM and an SI of 24.38 (Figure 3B); and in U937 cells with an IC_50_ of 170.6 μM and an SI of 7.95 (Figure 3C). The results demonstrate the effectiveness of MEL against different target cells during DENV infection.

### 3.4. MEL Acts on Post-Entry Step of DENV Infection

**Figure 4:**
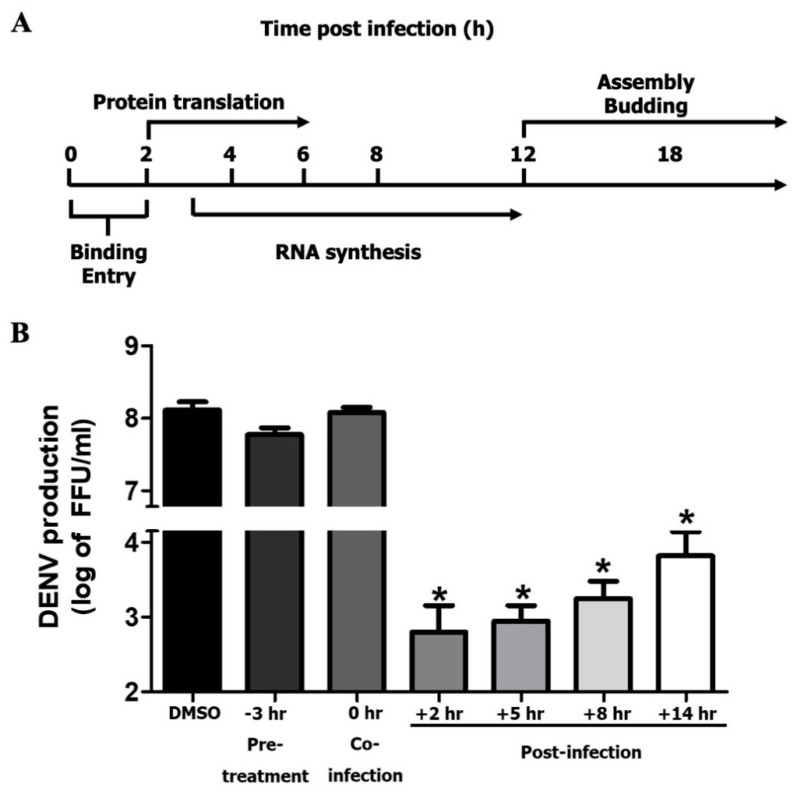
Time-of-addition study of MEL on DENV2-infected Huh7 cells. (**A**) Schematic timeline of DENV replication cycle. (**B**) Huh7 cells were treated with MEL prior to DENV2 infection (pre-treatment), during DENV2 infection (co-infection), or after infection (post-infection) at the indicated time-points. Culture supernatants were harvested at 24 h pi and viral titration was done. The asterisk (*) sign denotes *p* < 0.05 considered to be statistically significant.

**Figure 5:**
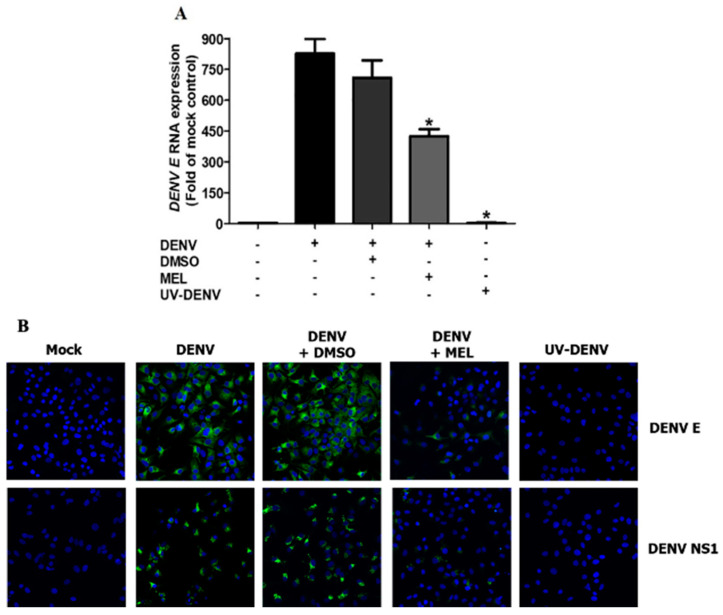
Effect of MEL on DENV RNA and protein expression. Huh7 cells were infected with DENV2 with and without UV-inactivation at MOI 1 and treated with vehicle control or 1000 µM MEL. (**A**) RNA and (**B**) protein expression was examined at 24 h pi by real time RT-PCR and IFA, respectively. Objective lens (40×) were used. The asterisk (*) sign denotes *p* < 0.05 considered to be statistically significant.

To study the effect of MEL on the timeline of DENV replication, a time-of-addition assay was performed. Figure 4A shows a timeline of the DENV life cycle, and Figure 5B shows the reduction in the viral titer that resulted from MEL treatment at the corresponding timepoints. Neither cell pretreatment (−3 h) nor cell co-treatment (0 h) with MEL before DENV infection influenced virus production, which suggests that MEL may not affect binding or entry of DENV into Huh7 cells. In contrast, post-treatment of Huh7 cells with MEL significantly inhibited DENV with observed decreases in the viral titer at +2, +5, +8, and +14 h. The viral titer was reduced from 7.21 × 10^8^ to 6.31 × 10^2^, 8.83 × 10^2^, 1.75 × 10^3^, and 6.55 × 10^3^ FFU/mL, respectively. This finding suggests that MEL may affect the post-entry stages of DENV infection.

### 3.5. MEL Interferes with Viral RNA and Protein Expression

To further clarify the result from the time-of-addition study, real-time RT-PCR and IFA experiments were performed to investigate the effect of MEL on viral RNA and protein expression. MEL treatment interfered with the kinetics of DENV E RNA expression over 24 h pi (Figure 5A). At 24 h pi, DENV E RNA expression was significantly lower in MEL-treated cells, compared to that of untreated cells. Similarly, DENV protein expression was attenuated by MEL, as demonstrated by a decrease in both DENV E positive and DENV NS1 positive cells upon treatment (Figure 5B). When taken together, these results suggest that MEL influences RNA synthesis and protein translation in the DENV life cycle.

### 3.6. Inhibition of the SIRT1 Reverses the Anti-DENV Effect of MEL

**Figure 6:**
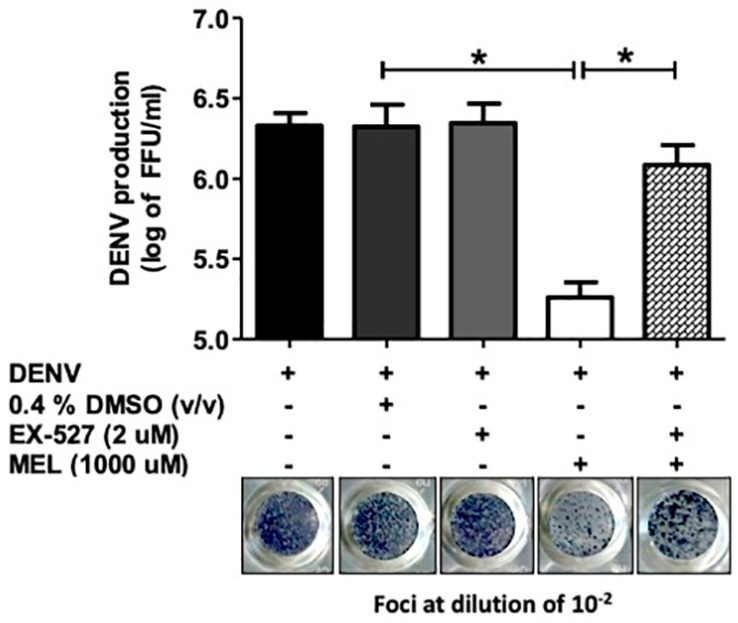
Effect of sirtuin 1 (SIRT1) inhibition with and without MEL treatment in DENV2-infected Huh7 cells. Huh7 cells were infected with DENV2 at MOI 1 and treated with the vehicle control or MEL with and without EX-527. Supernatants were collected at 24 h pi for virus titration. The asterisk (*) sign denotes *p* < 0.05 considered to be statistically significant.

To study the molecular mechanism of the anti-DENV effect of MEL, we evaluated the effect of MEL on DENV production, with and without SIRT1 inhibitor (EX-527). The CC_50_ value of EX-527 in Huh7 cells was 255.9 µM. EX-527 alone did not affect DENV production (Supplement Figure S1). We further examined the protein expression of MEL with and without EX-527 in DENV2- infected cells. When MEL was co-treated with EX-527, the protein expression of SIRT1 was reduced compared to that of the MEL treatment DENV-infected cell (Supplementary Figure S2). In the FFU assay, co-treatment of DENV-infected cells with MEL and EX-527 resulted in a viral titer of 1.2 × 10^6^ FFU/mL compared to a viral titer of 1.8 × 10^5^ in DENV-infected cells treated with MEL alone (Figure 6). This indicates that the antiviral activity of MEL is partly originated from its ability to activate the SIRT1 pathway.

### 3.7. MEL Enhances Antiviral IFN Response via the SIRT1 Pathway

**Figure 7:**
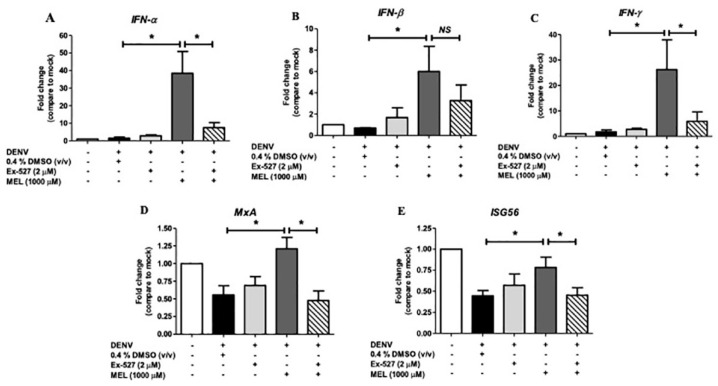
MEL enhances antiviral interferon (IFN) response via SIRT1 pathway. Huh7 cells were infected with DENV2 at MOI 1 and treated with MEL with and without EX-527. Cells were collected at 24 h pi and mRNA expression of antiviral genes was examined by real time RT-PCR. (**A**) IFN-α, (**B**) IFN-β, (**C**) IFN-γ, (**D**) MxA, and (**E**) ISG56. The asterisk (*) sign denotes *p* < 0.05 considered to be statistically significant.

Drugs that activate SIRT1 are known to inhibit the production of viral progeny of both DNA and RNA viruses. Host antiviral response, such as the IFN system and its downstream molecule ISG, is targeted and suppressed by DENV infection. We set forth to examine the effect of MEL, with and without the SIRT1 inhibitor, on genes of the IFN pathway. We investigated whether MEL reprieved the suppression and enhanced the antiviral response by examining the expression of antiviral genes downstream along the SIRT1 pathway. The expression of IFN-α, IFN-β, and IFN-γ was enhanced by MEL (Figure 7A–C). Furthermore, the effector molecules, including the MxA and ISG56 genes, that were downregulated in DENV infected cells, were upregulated upon treatment with MEL (Figure 7D,E). The upregulation of the expression of the IFN-α, IFN-γ, MxA, and ISG56 genes was significantly decreased upon co-treatment with MEL and EX-527. When taken together, these results suggest that the anti-DENV activity of MEL is partly mediated via the SIRT1 pathway and its downstream antiviral IFN pathway.

## 4. Discussion

Despite decades of research into DENV, no effective drug has been approved for clinical use during this viral infection. Drug repurposing has been shown to be a promising approach for discovering drugs to treat emerging viral infections around the world. In this study, we employed the drug repurposing approach, using MEL for inhibition of DENV infection. MEL is a hormone that is produced by the pineal gland in humans that regulates our sleep-wake cycle [20]. Exogenous MEL is safe for use with only mild side effects [21]. MEL is also well known for its antioxidant, anti-inflammatory, anti-excitatory, and immunoregulation properties [22,23,24,25]. MEL was used in experimental models of respiratory syncytial virus, Venezuelan equine encephalitis virus, viral hepatitis, viral myocarditis, and was recently proposed as a potential candidate treatment against severe acute respiratory syndrome coronavirus 2 (SARS-CoV-2) infection [9]. This led us to hypothesize that MEL may exert a protective antiviral outcome in DENV infection.

In order to determine a non-toxic concentration of MEL for our experiments, we conducted a cell cytotoxicity assessment (Figure 1A), and we used the non-toxic dose to study the anti-dengue effect in Huh7 cells. The SI, which is the ratio between cytotoxicity and the antiviral activity of the drug, was 23.49. Theoretically, the higher the SI ratio, the safer and more effective the drug should be when used to treat viral infection. One to ten milligrams of MEL are considered to be a standard dose for prescription as a sleeping pill [16]. The plasma concentration of MEL in healthy volunteers who were given 10 mg of MEL was 3550.5 (2500.5–8057.5) pg/mL [26]. In in vivo experiments, exogenously administered MEL was given in doses of up to 800 mg/kg without any severe toxic effects [27]. Similarly, in rats, doses of 200 mg/kg/day were administered during the gestation period and there was no report of increased prenatal mortality, change in weight of the fetus, or change in the rate of embryological deformities [28]. Our result demonstrated effective inhibition of virus production in MEL-treated conditions in a dose-dependent manner, and the observed inhibition exerted by MEL was stronger than that observed in NAC-treated DENV-infected cells (Figure 1B,C). Clinical studies in DENV infection demonstrated the role of serotype on dengue severity and clinical outcome [29]. In the present study, MEL reduced the production of all four serotypes of DENV; however, we observed variation in the level of inhibition among the four DENV serotypes (Figure 2). We further tested the effectiveness of MEL on EA.hy.926, A549, and U937 cells as representatives of infection in human endothelial, lung, and monocyte cell lines, respectively (Figure 3). MEL decreased DENV production in all cell types tested. However, a previous study reported that MEL affected DENV-infected HEK293T cell line, but did not affect DENV-infected HepG2 cell line [30]. This variation in outcome may be due to differences in the MOI of DENV used, the concentration of MEL used, and/or the method used to detect the effect. Whereas that previous study selected a specific concentration of MEL and used the percentage of DENV antigen-positive cells to detect the effect, our experiments focused on virus production capability as the indicator of the effectiveness of the drug.

To further elucidate the potential mechanism of DENV replication inhibition by MEL, we performed a time-of-addition study (Figure 4). We did not detect any change in the inhibition of DENV infection in pre-treatment or co-infection conditions, which suggests that MEL may not act on DENV itself or impede the DENV entry process. Alternatively, MEL likely interferes with the early post-entry phases of the DENV life cycle, since the inhibitory effect of MEL was observed only when it was added at 2 h pi up to 14 h pi. We therefore hypothesized that MEL may target either viral RNA replication, or, synthesis of viral proteins, thereby affecting DENV production. Our subsequent experiments narrowed the list of potential target stages of MEL to be the viral RNA and protein synthesis, since the addition of MEL resulted in reduced expression of DENV RNA and protein (Figure 5A,B). After examining the dose-dependent effect of MEL, we tested the effect of MEL at different timepoints of viral infection. Consistent with infectious virus produced at 24 h pi, we observed a marked decrease in the viral RNA and protein levels in MEL-treated DENV-infected cells. Our results suggest that MEL may act on the post-entry stage of the DENV life cycle.

MEL is well known for its ability to activate the sirtuin pathway by increasing the expression and activity of the SIRT1 protein [13,31]. SIRT1 is an evolutionarily conserved NAD^+^-dependent deacetylase that regulates the expression of other genes by deacetylating histones and transcription factors that include FOXO, NF-ĸB, P53, STAT3, and STAT1 [32]. Therefore, to examine the effect of MEL on the sirutin pathway, we examined the protein expression of SIRT1 in DENV-infected Huh7 cells with and without EX-527. EX-527 is a selective, cell-permeable inhibitor of SIRT1 (IC_50_ = 98 nM) and SIRT 2 (IC_50_ = 19.6 µM) [33]. In co-treatment of MEL and EX-527, we observed that SIRT1 expression was decreased when compared to MEL alone treatment (Supplementary Figure S2). Further, we examined the effect of MEL on DENV production with and without EX-527. When DENV-infected cells were co-treated with MEL and EX-527, virus replication was significantly restored when compared to MEL treatment alone, suggesting involvement of SIRT1-mediated mechanism in DENV production (Figure 6). Previously, SIRT inhibitors that inhibit both SIRT1 and SIRT2 are known to have antiviral properties in arboviral replication [34].

MEL can attenuate proinflammatory responses and inhibit apoptosis, which is mediated partly via the JNK, p38 mitogen-activated protein kinase (MAPK), NF-kB, and Nrf2 signaling pathways [35]. However, the effect of MEL on antiviral pathways remains unknown. Thus, to examine the effect of MEL on antiviral pathways, we assessed antiviral IFN expression. Our real-time RT-PCR experiments with IFN and ISGs further confirmed our hypothesis that MEL induced the IFN signaling pathway and the effector molecules (ISGs). Upon treatment with the SIRT1 inhibitor, the capability of MEL to trigger this antiviral signaling cascade decreased (Figure 7). This might be a possible mechanism of DENV inhibition in our experimental setting. IFN and ISGs both directly and indirectly inhibit viral infection by targeting various stages in the viral life cycle. Jiang, et al. found that the antiviral activity of type I IFN activates ISGs, which later disrupted multiple steps of DENV and West Nile virus infection [36]. MxA and ISG56 were previously reported to inhibit viral replication [37,38]. We also found significant increase in gene expression of IFN-α, IFN-β, and IFN-γ, and of the ISGs, MxA and ISG56 upon MEL treatment in DENV-infected cells, when compared to vehicle control. Upon addition of the SIRT1 inhibitor to MEL, the expression of these genes was significantly reversed, comparable to control. Both MEL and resveratrol [14] increased ISG expression and reduced DENV replication; therefore, it would be interesting to study if these two drugs exert a synergistic affect when used in combination.

**Figure 8:**
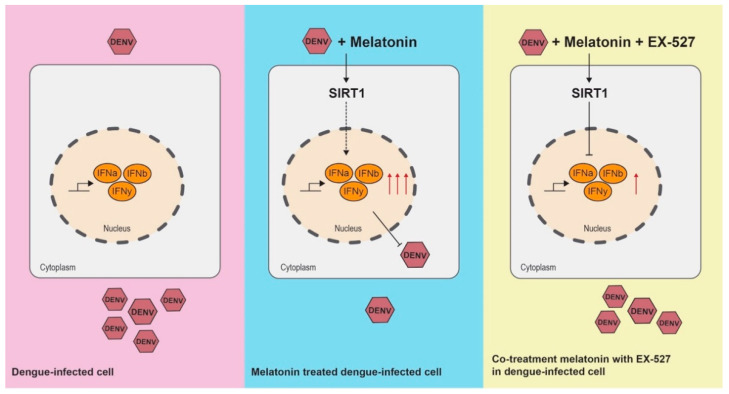
Proposed molecular mechanism of MEL in DENV-infected cells.

A proposed mechanism of action of MEL in DENV-infected cell is shown in Figure 8. More studies are needed to conclusively determine the mechanism of IFN activation in MEL-treated DENV-infected cells, and to optimize the concentration and potency of MEL.

## 5. Conclusions

DENV can replicate in Huh7 cells to produce new infectious virions. MEL treatment of DENV-infected cells can activate antiviral genes to inhibit viral replication; however, when the SIRT1 inhibitor is used in combination with MEL, the activation of these antiviral genes is decreased.

## Figures and Tables

**Table 1:** Primer sequence (5′ to 3′).

Primer	Forward	Reverse
DENV E	ATCCAGATGTCATCAGGAAAC	CCGGCTCTACTCCTATGATG
*IFN α*	TTTCTCCTGCCTGAAGGACAG	GCTCATGATTTCTGCTCTGAC
*IFN β*	GACGCCGCATTGACCATCTA	TTGGCCTTCAGGTAATGCAGA
*IFN γ*	ACTGACTTGAATGTGCAACGCA	ATCTGACTCCTTTTTCGCTTCC
*MxA*	ACCACAGAGGCTCTCAGCAT	CTCAGCTGGTCCTGGATCTC
*ISG56*	GGGCAGACTGGCAGAAGC	TATAGCGGAAGGGATTTGAAAGC
*GAPDH*	CGACCACTTTGTCAAGCTCA	AGGGGTCTACATCGCAACTG

## Supplementary Materials

The following are now available online at https://www.mdpi.com/article/10.3390/v13040659/s1, Figure S1: Effect of EX-527 on DENV2-infected Huh7 cells, Figure S2: SIRT1 protein expression in after MEL treatment in DENV2-infected Huh7 cells.
