# Supplementary material for: Melatonin Inhibits Dengue Virus Infection via the Sirtuin 1-Mediated Interferon Pathway
Source: Viruses. 2021 Apr 11;13(4):659. doi: 10.3390/v13040659 (PMC8070382; doi:10.3390/v13040659)
Supplement: Supplementary file 1 [file viruses-13-00659-s001.pdf]

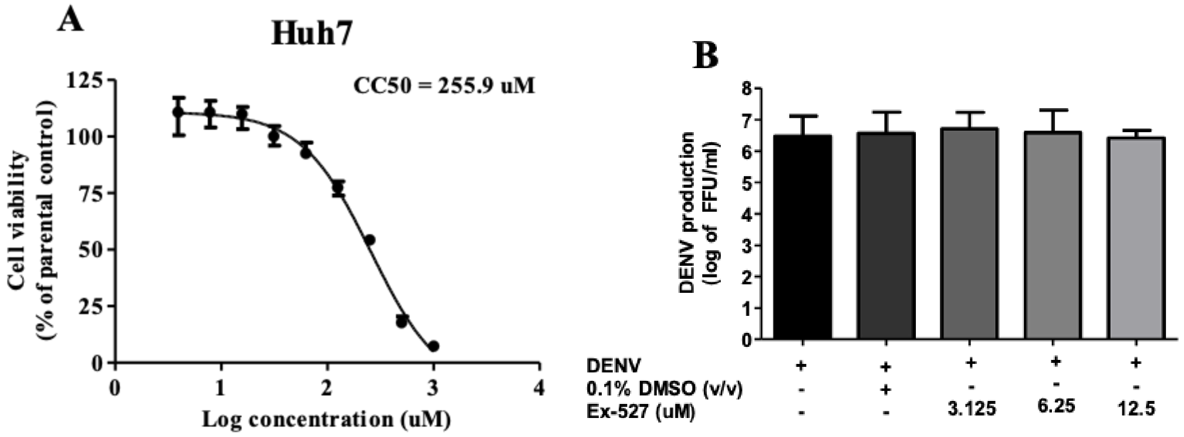

Figure S1.) Effect of EX-527 on DENV2-infected Huh7 cells. A) Huh7 cells were treated with EX-527 in a 2-fold serial dilution for 24 h. Cell viability was measured by Presto Blue assay and result was plotted into graph displaying the CC_{50} concentration. B) Huh7 cells were infected with DENV2 at MOI 1 and treated with EX-527 at various concentrations. Culture supernatant were collected at 24 h pi and FFU assay was performed.

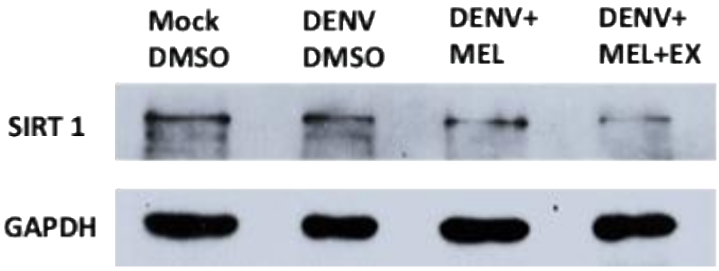

Figure S2.) SIRT1 protein expression after MEL treatment in DENV2-infected Huh7 cells. Huh7 cells infected with DENV2 at MOI1 and MEL was added with or without EX-527. SIRT1 protein expression was analyzed at 24 h pi by Western blot analysis. The intensities of the protein bands from experiment were normalized with GAPDH.
